# Safety of ipsilesional anodal transcranial direct current stimulation in acute photothrombotic stroke: implications for early neurorehabilitation

**DOI:** 10.1038/s41598-024-51839-5

**Published:** 2024-01-30

**Authors:** Brita Fritsch, Marleen Mayer, Janine Reis, Anne-Kathrin Gellner

**Affiliations:** 1 Department of Neurology, University Hospital Freiburg, Breisacher Str. 64, 79106 Freiburg, Germany; 2 Department of Psychiatry and Psychotherapy, University Hospital Bonn, Venusberg-Campus 1, 53127 Bonn, Germany

**Keywords:** Stroke, Cellular neuroscience

## Abstract

Early rehabilitation in the acute phase of stroke, that bears unique neuroplastic properties, is the current standard to reduce disability. Anodal transcranial direct current stimulation can augment neurorehabilitation in chronic stroke. Studies in the acute phase are sparse and held back by inconclusive preclinical data pointing towards potential negative interaction of the excitability increasing tDCS modality with stroke-induced glutamate toxicity. In this present study, we aimed to evaluate structural and behavioral safety of anodal tDCS applied in the acute phase of stroke. Photothrombotic stroke including the right primary motor cortex was induced in rats. 24 h after stroke anodal tDCS was applied for 20 min ipsilesionally at one of four different current densities in freely moving animals. Effects on the infarct volume and on stroke induced neuroinflammation were assessed. Behavioral consequences were monitored. Infarct volume and the modified Neurological Severity Score were not affected by anodal tDCS. Pasta handling, a more sensitive task for sensorimotor deficits, and microglia reactivity indicated potentially harmful effects at the highest tDCS current density tested (47.8 A/m^2^), which is more than 60 times higher than intensities commonly used in humans. Compared to published safety limits of anodal tDCS in healthy rats, recent stroke does not increase the sensitivity of the brain to anodal tDCS, as assessed by lesion size and neuroinflammatory response. Behavioral deficits only occurred at the highest intensity, which was associated with increased neuroinflammation. When safety limits of commonly used clinical tDCS are met, augmentation of early neurorehabilitation after stroke by anodal tDCS appears to be feasible.

## Introduction

Stroke is the worldwide leading cause of lasting motor impairment. The magnitude of spontaneous functional recovery is independent of external factors in the early days to weeks^1–4^. Next to spontaneous diminution of detrimental milieu factors in the stroke area (e.g., edema, inflammation) the acute brain injury opens a window for enhanced neuroplasticity. Enhanced reorganization on the network level is observable, similar to neurodevelopmental levels of plasticity^5–7^. Accordingly, an early start of rehabilitative treatment already in the acute setting of the stroke unit is an established procedure in stroke neurorehabilitation^8^. The definition of this early post-stroke period has not been clearly defined and varies between studies and guidelines, but is often agreed to include the first 7–14 days^9–11^. However, since high intensity rehabilitative activity increased infarct volume in animal studies^12–14^ and in some cases even worsened functional outcomes^14^ it is accepted that intervention intensity has to be adjusted to moderate levels. Increased infarct volume has been attributed to higher stroke related glutamate toxicity^12,13^.

Transcranial direct current stimulation (tDCS) modulates cortical excitability and synaptic plasticity across species in a polarity-dependent manner^15–17^. In healthy subjects and chronic stroke patients anodal tDCS promotes neuroplastic processes such as motor learning, enhanced upper limb function, and increased gray matter volume^18–22^. The underlying cellular effects contributing to behavioral improvements are well understood and include enhanced BDNF-signaling and improvement of synaptic transmission^23–26^. Thus, anodal tDCS application in conjunction with early neurorehabilitation could maximize utilization of the early plastic window after stroke, especially in patients with limited physical abilities. On the other hand, application of anodal tDCS in the acute phase may negatively interact with the stroke related cerebral environment and lead to delayed neurodegeneration, e.g., by glutamate excitotoxicity, as was shown for high intensity rehabilitative activity. Current animal studies of acute ipsilesional anodal tDCS after stroke were performed under anesthesia^27–30^. Anesthesia suppresses glutamate release^31,32^ and also tDCS induced cortex-wide Ca^2+^-elevations^33^ and detrimental effects of high intensity tDCS such as neuroinflammation and neurodegeneration^34^. Thus, anesthesia likely masks harmful effects of acute tDCS in stroke. Moreover, with the exception of one study^27^, stimulation intensities in rodent studies were manifold above those used in clinical application. Nevertheless, the insulating structures (e.g., bone) are much thinner in rodents compared to the human skull and thus rodent studies are likely overestimating harmful effects of the stimulation. While there is also some evidence from modeling studies on how the parameters could possibly relate^35^, the optimal stimulation parameters per species may differ. Under these technical conditions with limited transferability to the human condition, only one study demonstrated an increase in infarct volume through anodal tDCS^29^ while in the other studies infarct volume was not affected by tDCS^27,28,30^.

In the present study, using a rat stroke model with consistent infarct location and size (via photothrombosis) we evaluate safety aspects of acute ipsilesional anodal tDCS in a dose–response design. One day after stroke induction anodal tDCS was applied to non-anesthetized rats at one of four different stimulation intensities, including those leading to slight neuroinflammation in the non-stroke rat brain^34^. Infarct volume and microglial reactivity were investigated, as well as effects on the behavioral level measured by the modified Neurological Severity Score and the pasta handling task. We hypothesize that low intensity ipsilesional anodal tDCS can be safely applied early post stroke in rats.

## Methods

### Animals

Adult male Sprague Dawley rats (n = 29, age 8.5 ± 0.5 weeks, 309–400 g; Charles River, Sulzfeld, Germany), were group-housed in standard cages on a 12/12 h light/dark cycle at constant room temperature. Animals had access to water and food ad libitum, except 24 h prior to testing, when the food was weight-adjusted (10 g/100 g body weight). All animal studies were performed according to the Animal Protection Law and Directive 2010/63/EU of the European Commission. Animal protocols were approved by the Commission for Animal Experimentation of the Regional Council of Freiburg and the Commission for Animal Experimentation of the University Medical Center Freiburg. All animal experiments have been reported in compliance with the ARRIVE guidelines.

**Figure 1:**
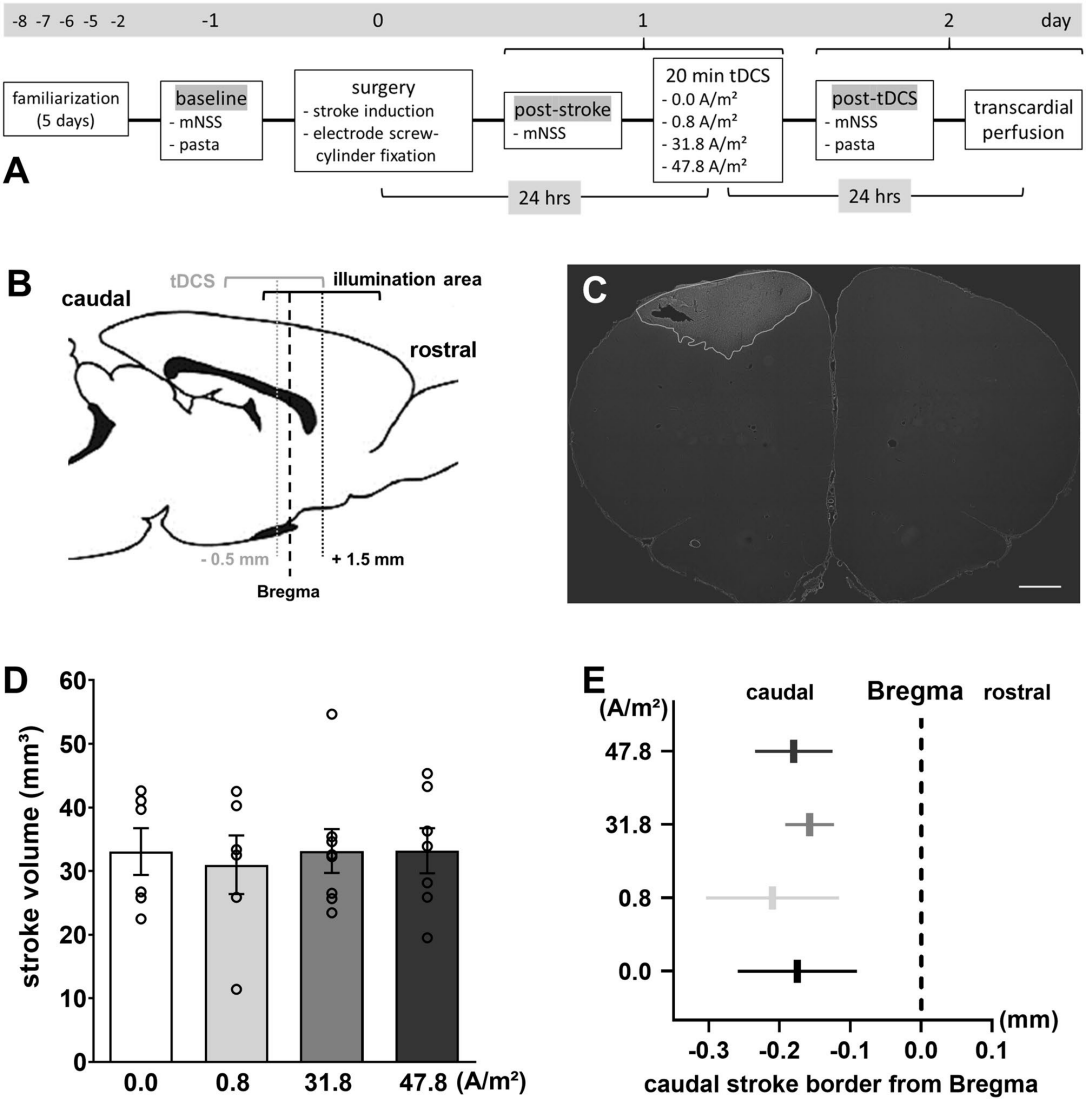
Experimental overview and lesion data. (**A**) Experimental timeline. (**B**) Schematic sagittal rat brain drawing depicting the tDCS area and the illumination area (photothrombotic stroke induction); midpoint of the areas in relation to bregma (dotted lines). (**C**) Representative image of a FJ-C**-**stained histological brain slice; stroke delineated in white; 1000-µm scale bar. Photothrombosis resulted in comparable (**D**) infarct volume and (**E**) extension of the caudal stroke border, independent of the group. 0.0 A/m^2^ (n = 6), 0.8 A/m^2^ (n = 5), 31.8 A/m^2^ (n = 8), 47.8 A/m^2^ (n = 7). *mNSS* modified neurological severity score, *pasta* pasta handling task, *tDCS* transcranial direct current stimulation. Data represent mean ± SEM, scatter = individual animals.

After receiving stroke surgery, rats were assigned to the four different treatment groups (sham tDCS = 0.0 A/m^2^, anodal tDCS with either 0.8 A/m^2^, 31.8 A/m^2^ or 47.8 A/m^2^) by a stratified step-by-step randomization performed by the lead investigator to maintain comparable post-stroke modified neurological severity scores per treatment arm. Stimulation intensities were based on our previous study, which found the threshold for neurodegeneration to be at or below 31.8 A/m^2^ in anesthetized or awake rats^34^. The experimental timeline is depicted in Fig. 1A. Recruitment proceeded until each group included at least 5 rats, extrapolated as an appropriate number of animals for analysis based on the effect sizes in a similar dose–response experiment in healthy rats^34^. The investigator performing the behavioral testing as well as data analysis was blinded for the type of tDCS applied.

### Stroke induction

Ischemic stroke was induced via photothrombosis as described elsewhere^36^. In brief, under isoflurane anesthesia (4% induction, 1.5–2% maintenance), Bengal rose (Sigma Aldrich, St. Louis, MO, USA; 9.975 mg/kg; dissolved in isotonic saline) was injected into the lateral tail vein of the stereotaxically fixed rat. With the beginning of the injection (slowly applied over 2 min) a circular area targeting a cortical region including the left primary motor cortex (5 mm diameter: center relative to Bregma: anterior–posterior =  + 1.5 mm, medial–lateral = 3.5 mm left, Fig. 1B) was illuminated with a cold light source (KL 1500 LCD 150 W, SCHOTT AG, Mainz, Germany) through the exposed skull and illumination continued for a total of 20 min.

### Transcranial DCS

Immediately following the cold light illumination, the DCS electrode was placed during the same surgery session. A plastic cylinder was fixed with acrylic cement to two screws on the skull (inner diameter: 4 mm, center relative to Bregma: anterior–posterior = − 0.5 mm, medial–lateral = 3.5 mm left, Fig. 1B). Photothrombosis and tDCS areas had only a partial overlap, resulting in brain regions underlying the DCS electrode only (most caudal; region D in Fig. 3B), underlying both the DCS electrode and the photothrombosis (mid-section; region E in Fig. 3B), and most rostral underlying the photothrombosis only (region F in Fig. 3B).

In a separate session, tDCS was performed 24 h after stroke induction in the awake, freely moving animals. After filling the cylinder with isotonic saline solution, the tDCS electrode (Ag/Ag-Cl, contact area of 12.56 mm^2^; World Precision Instruments, Sarasota, FL, USA and in-house production) was screwed in. A rubber counter electrode (6 cm^2^) was covered with conductive gel and attached to the animal's chest with a vest.

Stimulation parameters were set to current densities of either 0.0, 0.8, 31.8 or 47.8 A/m^2^, and current was turned on for 20 min using a 9 V battery-driven stimulator with a one second ramp-up and -down function (in-house production, Technical Workshop, Department of Neurology, University of Freiburg). During stimulation, the behavior of the animal and the current flow were constantly monitored.

### Familiarization

Rats were familiarized to all procedures performed during alertness. This included the tDCS set-up (wearing the vest containing the chest electrode, cage), traversing a round beam (length 1 m, diameter 2.5 cm) to reach a dark box included in the modified Neurological Severity Score (mNSS) and handling the pasta after a brief 24 h weight-adjusted food restricted period (10 g/100 g body weight)*.*

### Modified neurological severity score

Neurological deficits after stroke were assessed by the mNSS with a potential maximum deficit score of 18^37^. Scores were assessed at baseline (day -1), post-stroke (day 1) and post-DCS (day 2, timeline see Fig. 1A). In brief, sensorimotor testing included placing of the affected forepaw on a surface after the vibrissae were touching it (placing test, 0–1 point) and contraction of the forepaw while pushing it to a surface (proprioceptive test, 0–1 point). Three repetitions of the beam balance test were averaged (steady balancing to falling off, 0–6 points). For further motor testing, walking (normal walk to falling to the paretic side, 0–3 points) and the reaction to raising by the tail (1 point each for flexion of fore- or hindlimb within 60 s or turning of the head > 10° to vertical axis within 30 s), were observed. Missing corneal, pinna or startle reflexes or any seizure-like abnormalities were scored with 1 point each.

### Pasta handling task

Fine motor performance was assessed in rats handling and eating a piece of pasta (length 7 cm, diameter 1.5 mm^38,39^)*.* Rats were placed in the experimental cage. One pasta piece was handed at a time. Testing concluded after a maximum of five pasta pieces, or after 4 valid trials. This resulted in 1–4 valid trials per rat for the final analysis. Single performances per animal were averaged. Reasons for invalid performances were no performance at all or pasta breaks, which alter the difficulty of the task^39^. Performance was video-taped for offline analysis (60 frames/s). For frame-by-frame analysis Avidemux software (Version 2.6.12, http://fixounet.free.fr/avidemux/) was used. General performance was assessed by the time needed to eat each piece of pasta. Asymmetries in limb use were assessed by counts of ipsi- and contralateral grasps per pasta (in relation to the stroke hemisphere). In addition, each atypical behavior was counted as either present (1 point) or non-present (0 points). Atypical behaviors: hunched posture*,* angling with head tilt, paws together when long, paws apart when short, guide and grasp switch, mouth pulling*.* The category failure to contact was divided into 3 separate categories:*—*affected paw supports, no grip,—no contact with affected paw during eating (contact during adjustment excluded)*,—*no contact with affected paw at any time. Because of the inability of most animals to perform the task 1 day after stroke induction, poststroke-data were not analyzed.

### Tissue preparation

24 h after tDCS, rats were deeply anesthetized with ketamine (80 mg/kg body weight) and xylazine (12 mg/kg body weight) intraperitoneally and, following a negative toe pinch reflex, perfused transcardially with cold phosphate-buffered saline pH 7.4 (PBS) followed by 4% paraformaldehyde dissolved in PBS (PFA). The brains were harvested, postfixed in 4% PFA at 4 °C for 24 h and cryoprotected at 4 °C in 30% sucrose for 48 h. Afterwards, the brains were cut into coronal sections of 30 μm using a freezing microtome.

### Immunohistochemistry

For detection of degenerated neurons, brain sections were mounted on gelatine-coated slides, allowed to dry, and stained with Fluoro-Jade C (FJ-C, Merck Millipore, Darmstadt, Germany) as described elsewhere^40^. Slides were successively incubated in solutions of 1% NaOH in 80% ethanol (5 min), 70% ethanol (2 min), distilled water (2 min), 0.06% potassium permanganate (10 min), distilled water (2 min) and 0.0001% FJ-C staining solution (10 min, 0.01% FJ-C stock solution diluted in 0.1% glacial acetic acid). After washing (3 × for 1 min in distilled water) and drying, slides were cleared in xylene and cover slipped with DPX (Thermo Fisher Scientific, Waltham, MA, USA).

For visualization of microglia selected brain sections of three different regions (anterior–posterior from Bregma: + 3.8 to + 4.5 mm (only photothrombosis, Fig. 3B,F); Bregma − 0.1 to + 0.2 mm (photothrombosis and tDCS, Fig. 3B,E) and Bregma − 2 to − 1.7 mm (only tDCS, Fig. 3B,D) were incubated with blocking solution (3% donkey serum in 0.1% PBS-Tween) for 30 min at room temperature, then incubated at 4 °C for 24 h with the primary antibody rabbit against ionized calcium-binding adapter molecule 1 (anti-Iba1; 1:1000, #019-19741, Wako Chemicals, Neuss, Germany) diluted in 3% donkey serum and 5% bovine serum albumin in 0.1% PBS-Tween. Afterwards, sections were washed (3 × in 0.5% PBS-Tween for 10 min) and incubated for 60 min protected from light in the secondary antibody Alexa Fluor 555 (1:500 in 0.5% PBS-Tween, Molecular Probes, Thermo Fisher Scientific, Waltham, MA, USA). DAPI-staining (1:1000 in PBS, Sigma Aldrich, St. Louis, MO, USA) was performed as a nuclear counterstain in between two washing steps in PBS. Brain sections were then mounted on slides, allowed to dry overnight and cover slipped with ProLong Gold Antifade Mountant (Thermo Fisher Scientific, Waltham, MA, USA).

### Image analysis

The lesioned and contralesional cortex were imaged using the GFP- (FJ-C, 480 ± 15 nm) and TexasRed-filter (anti-Iba1; 560 ± 20 nm) of Keyence BZ-9000 and BZ-X710 fluorescence microscope. Fiji software^41^ was used for image analysis.

Stroke volume was measured by truncated cone calculation of tagged FJ-C-stained areas (Fig. 1C), factoring in the sagittal distance between each brain section^42^. The maximum caudal stroke extension towards the region underlying the DCS electrode was assessed in relation to Bregma.

Perilesional cortical intensity of anti-Iba1-staining as a marker for microglial reactivity was measured by the mean gray values of 200 × 200 µm tiles (Matlab R2012b-Software, The MathWorks, Inc., Natick, MA, USA) and compared to the corresponding area of the contralesional hemisphere. The stroke area and artifacts were manually excluded from the analysis (Fig. 3A). The occurrence of high microglia reactivity was classified as 1.5 SD above the mean gray value of the non-lesioned hemisphere and utilized for analysis.

### Statistical analysis

**Figure 2:**
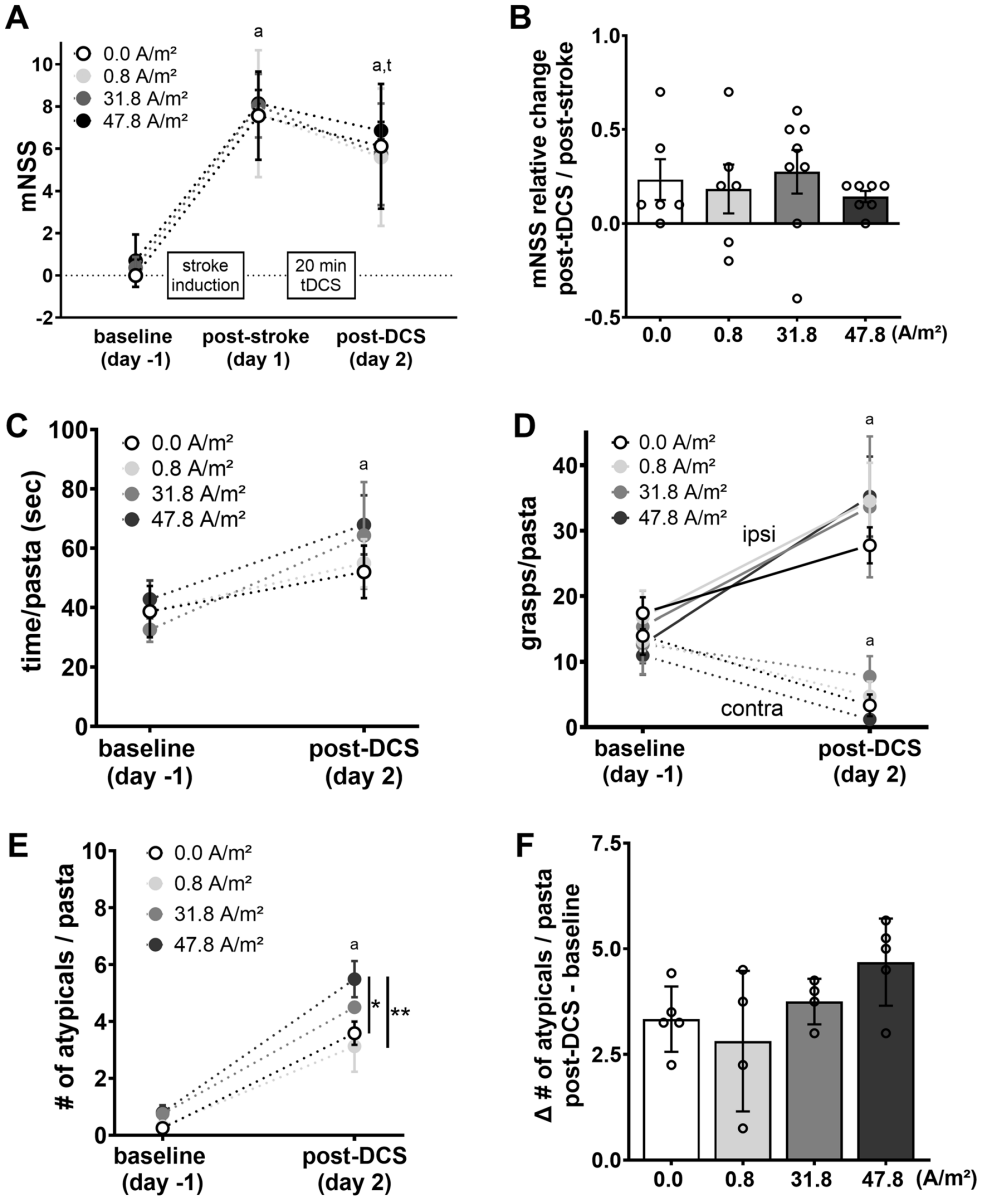
Effects of tDCS on neurological and behavioral deficits after photothrombotic stroke. (**A**) Across groups stroke led to a neurological impairment reflected by an increase in mNSS scores at post-stroke (day 1), followed by a slight decrease post-DCS (day 2). (**B**) The relative mNSS changes from (day 1) to (day 2) did not differ between stimulation groups. (**C**) Stroke resulted in general impairment in the pasta handling task assessed by the time needed per pasta. (**D**) Post-intervention contralateral forelimb impairment as indicated by asymmetrical distribution of grasps per pasta between limbs across all groups. (**E**) Stroke increased atypical behavior counts per pasta in all groups. The 47.8 A/m^2^ DCS group showed the highest counts on (day 2). (**F**) Analysis of atypical behavior counts from (baseline) to (day 1) revealed no difference between stimulation modalities. mNSS: 0.0 A/m^2^ (n = 6), 0.8 A/m^2^ (n = 5), 31.8 A/m^2^ (n = 8), 47.8 A/m^2^ (n = 7); pasta handling task: 0.0 A/m^2^ (n = 5), 0.8 A/m^2^ (n = 4), 31.8 A/m^2^ (n = 4), 47.8 A/m^2^ (n = 5). *mNSS* modified neurological severity score, *tDCS* transcranial direct current stimulation, *atypicals* atypical behaviors. Data represent mean ± SEM, scatter = individual animals. ^a^Different from baseline (day -1); ^t^trend towards difference from post-stroke (day 1), *p < 0.05, **p < 0.01.

**Figure 3:**
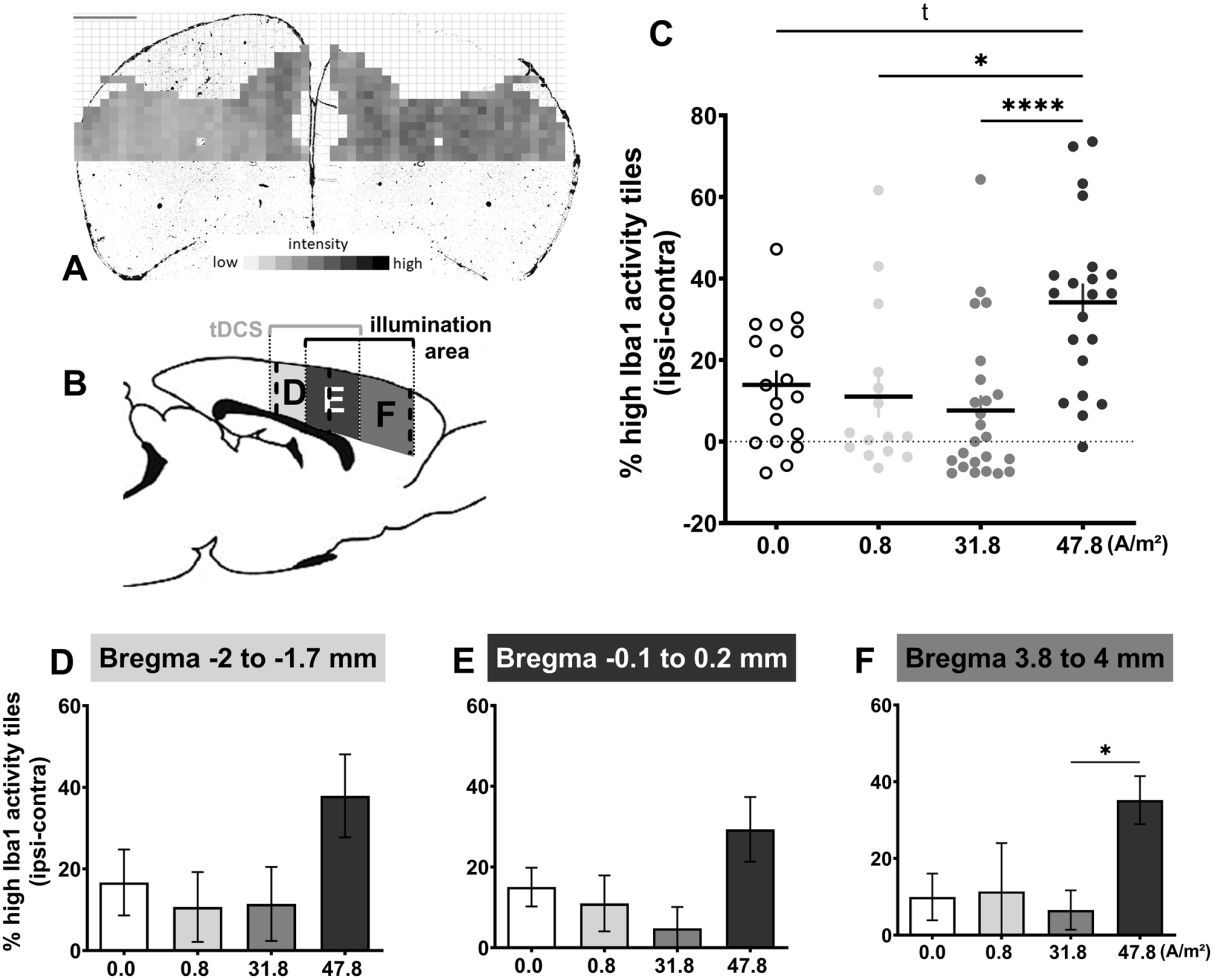
Effect of tDCS 24 h after photothrombotic stroke on microglia activation (**A**) Exemplary depiction of the 200 × 200 µm tiles utilized for analysis of high Iba1-reactivity, taken from region F in (**B**). Note that the non-lesioned hemisphere (left side on the figure) defined the corresponding tiles on the lesion side (right). (**B**) Schematic drawing of a rat brain in the sagittal plane with projection of the region underlying tDCS only (**D**; light grey), tDCS and photothrombosis (**E**; dark grey), and photothrombosis only (**F**; medium grey), and the location of the analyzed slices within the region (dotted lines). (**C**) The ratio of high Iba1-reactivity tiles across all regions was highest with 47.8 A/m^2^ tDCS and comparable between the other stimulation groups (ratio of high reactivity tiles per hemisphere, contralesional subtracted from ipsilesional). (**D**–**F**) Separation of the data corresponding to the regions depicted in (**B**) reflects this pattern for 47.8 A/m^2^ in each region, particularly in region F (photothrombosis only) vs. 31.8 A/m^2^. 0.0 A/m^2^ (n = 6), 0.8 A/m^2^ (n = 5), 31.8 A/m^2^ (n = 8), 47.8 A/m^2^ (n = 7). *tDCS* transcranial direct current stimulation. Data represent mean ± SEM, scatter in C = individual animal data points sampled across the 3 regions. ^t^trend, *p < 0.05, ****p < 0.0001.

All statistical analyses were performed using GraphPad Prism version 9.1.1 (GraphPad Software, San Diego, California USA). Normal distribution was tested by Shapiro–Wilk’s test. Data were either compared using a one-way ANOVA to describe group effects (Figs. 1D, 2B,F, 3C–F) or 2-way repeated measures ANOVA (Fig. 2A,C–E) to describe group and time effects as well as their interaction, followed by post-hoc Tukey’s test corrected for multiple comparisons. Not normally distributed data were compared using the Wilcoxon test (2 groups) or the Kruskal–Wallis test (> 2 groups) with Dunn’s corrected post-hoc test. For non-parametric repeated measures analysis Friedman test was used.

For all statistical comparisons, the level of significance was set to *p* < 0.05. All data are presented as mean ± standard error of mean (SEM), additional individual data are shown as scatter plots where appropriate.

## Results

Three rats were excluded from the experiment: one animal had a lack of stroke, one showed aberrant stroke location, and one could not be sufficiently familiarized with the procedures due to fearful behavior, resulting in final group sizes of 0.0 A/m^2^ n = 6, 0.8 A/m^2^ n = 5, 31.8 A/m^2^ n = 8, 47.8 A/m^2^ n = 7.

### Stroke volumes

Photothrombosis induced an ischemic lesion in the forelimb sensorimotor cortex region (Fig. 1C). Histological assessment revealed stroke volumes of 33.1 ± 3.67; 30.1 ± 4.61; 33.1 ± 3.45 and 33.19 ± 3.54 mm^3^ for 0.0, 0.8, 31.8 and 47.8 A/m^2^ tDCS, respectively, without any significant difference between groups (F(3, 23) = 0.07; P = 0.97, Fig. 1D). The caudal stroke border was underlying the tDCS electrode and caudal extension might be affected by the stimulation. The location of the caudal lesion border was most caudal with 0.8 A/m^2^ (− 0.21 mm ± 0.09 from bregma) and least caudal with 31.8 A/m^2^ (− 0.16 mm ± 0.03) but did not differ between stimulation groups (H(3) = 0.19; P = 0.97, Fig. 1E).

### Time course of mNSS

At baseline mNSS did not differ between groups (H(3) = 6.57, P = 0.87; Fig. 2A). Following stroke induction, in all 4 groups the mNSS increased significantly post-stroke to 7.56 ± 0.85; 7.66 ± 1.34; 8.04 ± 0.53; and 8.14 ± 0.24 for 0.0, 0.8, 31.8 and 47.8 A/m^2^ tDCS, respectively. mNSS remained elevated post-tDCS 6.11 ± 1.21; 5.6 ± 1.46; 5.74 ± 0.85; and 6.86 ± 0.16 (groups see above, increasing stimulation intensity). There was an effect of time on mNSS (Q(3) = 46.72; p < 0.0001, Fig. 2A), due to significant higher scores post-stroke (day 1) and post-DCS (day 2) vs. baseline and a trend of a decline from day 1 to day 2. Analysis of the relative change in mNSS from post-stroke (day1) to post-tDCS (day 2) did not differ significantly between stimulation groups (0.0 A/m^2^ = 0.23 ± 0.11 0.8 A/m^2^ = 0.18 ± 0.13; 31.8 A/m^2^ = 0.28 ± 0.11; 47.8 A/m^2^ = 0.14 ± 0.03; H(3) = 2.08, P = 0.55; Fig. 2B).

### Time course of pasta handling performance

8 rats had to be excluded from pasta handling analysis because of “no performance” or “breaks pasta”. In detail no performance occurred already at baseline in 1 animal with 31.8 A/m^2^, and 47.8 A/m^2^ resulting in following group sizes 0.0 A/m^2^ n = 6, 0.8 A/m^2^ n = 5, 31.8 A/m^2^ n = 7, and 47.8 A/m^2^ n = 6. The stroke in combination with tDCS lead to further exclusions due to “no performance” of 3 animals after 31.8 A/m^2^ and 1 after 47.8 A/m^2^, while 0.0 and 0.8 A/m^2^ did not. But, in each of the latter 2 groups 1 animal had to be excluded due to frequent pasta breaks.

Stroke impaired pasta handling was not affected by stimulation in so far that the time needed per pasta similarly increased over time in all groups (0.0 A/m^2^: from 38.67 s ± 8.62 to 52.01 s ± 8.84 ; 0.8 A/m^2^: from 38.76 s ± 1.85 to 54.94 s ± 8.12; 31. 8 A/m^2^: from 32.61 s ± 4.12 to 64.32 s ± 17.99 and 47.8 A/m^2^: from 42.81 s ± 6.34 to 67.85 s ± 10; TIME: F (1, 14) = 13.89, p = 0.0023; Stimulation: F (3, 14) = 0.41, p = 0.75; Time × Stimulation: F (3, 14) = 0.52, P = 0.68; Fig. 2C). Specifically, utilization of the contralateral limb for the pasta handling task was impaired after stroke and tDCS, reflected in a significant reduction of contralateral grasps per pasta from baseline to day 2 (0.0 A/m^2^: from 13.96 ± 2.89 to 3.35 ± 1.65 ; 0.8 A/m^2^: from 13.13 ± 1.48 to 4.75 ± 2.25; 31.8 A/m^2^: from 12.69 ± 4.57 to 7.75 ± 3.12 and 47.8 A/m^2^: from 10.96 ± 2.95 to 1.2 ± 0.58; W(3) = 135.0, p = 0.0005; Fig. 2D). The contralateral limb grasps on day 2 were not affected by the stimulation as the analysis of the absolute grasp difference from baseline to day 2 (post-stroke and -tDCS) did not differ significantly between groups (F (3, 14) = 0.29, p = 0.83). As expected, this was accompanied by a significant increase of grasps on the unaffected ipsilateral limb in all animals (0.0 A/m^2^: from 17.4 ± 2.43 to 27.76 ± 2.74; 0.8 A/m^2^: from 16.75 ± 4.09 to 34.52 ± 5.87; 31.8 A/m^2^: from 15.38 ± 5.43 to 33.63 ± 10.78 and 47.8 A/m^2^: from 12.65 ± 2.86 to 35.2 ± 6.11; TIME: F (1, 14) = 27.52, p = 0.0001; Fig. 2D) independent of the stimulation group (GROUP: F (3, 14) = 0.09, p = 0.96) and without STIMULATION x TIME interaction (F (3, 14) = 0.67, p = 0.59).

Additionally, occurrence of atypical behaviors increased after stroke and tDCS on day 2 (0.0 A/m^2^: from 0.25 ± 0.14 to 3.58 ± 0.40; 0.8 A/m^2^: from 0.31 ± 0.19 to 3.13 ± 0.89; 31.8 A/m^2^: from 0.75 ± 0.18 to 4.5 ± 0.20 and 47.8 A/m^2^: from 0.8 ± 0.26 to 5.48 ± 0.64, TIME: F (1, 14) = 208.9, p < 0.0001; Fig. 2E) with a significant difference between groups (GROUP: F (3, 14) = 3.46, p = 0.0454). As can be seen in Fig. 2E this effect was driven by the 47.8 A/m^2^ group with the highest atypical count on day 2 (vs. 0.0 A/m^2^ p = 0.0151, and vs. 0.8 A/m^2^ p = 0.0038). However, there was only a trend towards a STIMULATION x TIME interaction (F (3, 14) = 2.55, p = 0.097). Correspondingly, analysis of the absolute difference in atypical behavior counts from baseline to day 2 revealed only a slight trend towards a stimulation effect (F (3, 14) = 2.55, p = 0.097, Fig. 2F).

### Effect of tDCS on post-stroke microglia activation

Across all analyzed brain regions (Region D + E + F, Fig. 3A,B) high microglial reactivity was similarly expressed 24 h after 20 min of the lower tDCS intensities 0.0, 0.8 and 31.8 A/m^2^ tDCS, that was 48 h post-stroke (0.14 ± 0.04, 0.11 ± 0.05, and 0.08 ± 0.38, respectively; Fig. 3C). The highest tDCS intensity, 47.8 A/m^2^, resulted in a significant increase of high Iba1-reactivity (0.34 ± 0.05; H(3) = 20.29, p = 0.0001; p = 0.0554 vs. 0.0 A/m^2^, p = 0.0116 vs. 0.8 A/m^2^, p < 0.0001 vs. 31.8 A/m^2^; see Supplementary Fig. S1 for representative images of low and high microglial reactivity). Assuming spatial specificity of the tDCS effect on the underlying brain, stimulation and stroke areas were chosen so that there was only a partial overlap. This led to brain areas underlying the tDCS electrode caudal to the stroked brain region, underlying the tDCS electrode and being affected by stroke, and being affected by stroke while not underlying the tDCS electrode (Fig. 3B). As can be seen in Fig. 3D–F, the proinflammatory effect of high-dose 47.8 A/m^2^ tDCS occurred in all three brain regions. In region F a significant group difference was found (H(3) = 8.93, p = 0.030, p = 0.14 vs. 0.0 A/m^2^, p = 0.19 vs. 0.8 A/m^2^, p = 0.0442 vs. 31.8 A/m^2^; Fig. 3F). In region E a trend (H(3) = 6.46, p = 0.09, Fig. 3E), and no statistical difference in region D (H(3) = 5.92, p = 0.12, Fig. 3D) were found.

## Discussion

Anodal tDCS applied at low stimulation intensity to the ipsilesional forelimb motor cortex 24 h after photothrombotic stroke in awake rats, is safe as assessed by stroke lesion size, microglial reactivity, neurological deficits**,** and sensorimotor performance. This is the first study investigating the safety limits of acute anodal tDCS in a dose-dependent manner and in freely moving rodents with a stroke. Indicators for dose-dependent harmful effects of anodal tDCS were found at stimulation intensities clearly exceeding those used in clinical application (i.e., typically 0.4–0.8 A/m^2^).

Previous studies in rodent models were inconclusive with regard to the influence of anodal tDCS in the acute phase after stroke on stroke lesion size in rodents^27–30^. A single study reporting an increase in infarct size in mice^29^ used a much earlier tDCS application (during/within 4.5 h of stroke induction) compared to the others, which had at least a 24 h delay after stroke and were performed in rats. In line with the latter studies, low to high intensity anodal tDCS applied with a delay of 24 h after stroke induction in our present study did not affect stroke volume in awake rats. Partial placement of the tDCS electrode behind the photothrombotic area allowed us to additionally assess whether tDCS influences the caudal infarct border, which might indicate harmful effects that might be otherwise masked in the overall stroke volume. In anesthetized healthy rats, neurodegeneration was detectable already at 47.8 A/m^2^ (> 60 times higher than in the human application), with the extent even pronounced in the absence of anesthesia^34^. However, since in the present stroke study additional neurodegeneration to the stroke volume was not detectable at the threshold intensity in non-stroke rats^34^, it is conceivable that there is no negative interaction between stroke and tDCS. The apparently lower lesion threshold in non-stroke rats in the previous study is likely explained by the high sensitivity of the FJ-C-stain in the healthy brain, where even a single degenerating neuron can be identified, and the threshold can be set with very high sensitivity. In fact**,** at 47.8 A/m^2^ only sparse isolated degenerating neurons were found in healthy rats. Such a finding could likely be obscured within the stroke induced neurodegeneration. We did not test higher intensities in stroke rats to find the lesion threshold under this condition as we intended to perform the stimulation in wakefulness. In our hands 47.8 A/m^2^ is the highest intensity well tolerated, judged by behavior (head scratching).

On the behavioral level, the rats’ neurological status improved relative to the first assessment post-stroke, regardless of the tDCS intervention. This finding is in line with previous work observing no aggravation of post-ischemic neurological or behavioral deficits after anodal tDCS to the stroke hemisphere in anesthetized rats^27–29^. Interestingly, despite the increased infarct volume after hyperacute ipsilesional high intensity (55.5 A/m^2^) anodal tDCS in mice, there was no behavioral correlate as measured by the mNSS^29^. As the mNSS covers several neurological functions (motor, sensory, reflexes and balance), there is a possibility that specific deficits in one function may be masked. Here, we used the pasta handling task to additionally assess manual skillfulness and in particular motor and sensory dysfunction with a task more sensitive to reveal even subtle deficits. Indeed, a group difference in atypical behaviors with more frequent occurrence after 47.8 A/m^2^ compared to 0.0 and 0.8 A/m^2^ was present. While this finding was not reflected in a time x stimulation interaction, it is one hint towards a negative effect of anodal tDCS on behavioral function at this particular stimulation intensity. Of note, drop-outs due to “no performance” only occurred at the two highest tDCS intensities. The lack of harmful effects of lower intensity ipsilesional anodal tDCS in our study supports two small monocentric clinical trials in acute stroke patients that did not find negative effects on behavioral outcome measures (structural assessment was not performed)^43,44^.

Neuroinflammation, indicated by increased microglia reactivity, occurred with high intensity anodal tDCS in healthy rats^34,45,46^. With increasing stimulation intensity microglia activation preceded neurodegeneration and might thus be a more sensitive marker than neurodegeneration to detect harmful tDCS effects^34^. Additionally, it is well known that microglia are activated in response to cerebral ischemia, which might be mediated by increased glutamate or cytokine release from affected neurons. As expected, based on our previous results in healthy rats, high intensity tDCS applied at 47.8 A/m^2^ resulted in higher stroke related microglia activation in contrast to sham or lower intensity tDCS at 0.8 and 31.8 A/m^2^. This effect was homogenously distributed across the differently exposed brain regions (see Fig. 3B,D–F). Since the intensity threshold for microglia activation was not reduced compared to the healthy rat exposed to the same stimulation procedure^34^, one can argue that the susceptibility of the rat brain to anodal tDCS-induced neuroinflammation is not higher in the early days after stroke. In accordance, high intensity anodal tDCS failed to induce potentiation of ischemia related microglia activation in anesthetized rats^29,30^. On the other hand, low intensity anodal tDCS may even protect from post-ischemic axonal degeneration in the internal capsule^27^.

While we have not assessed the effects of cathodal tDCS in our study, it is important to note that in preclinical studies cathodal tDCS seems to have neuroprotective effects after stroke^29,47–49^, most notable even when applied in the hyperacute phase^29,47,48^, a finding that has been ascribed to its excitability-reducing effects and a consecutive attenuation of stroke-induced glutamate toxicity^29,47^. A recent clinical pilot study demonstrated safety and feasibility of cathodal tDCS in the hyperacute phase of stroke. Only a trend was found towards reduced infarct growths in the first 24 h, paving the grounds for future trials^50^. Additional data from a clinical trial regarding the safety, tolerability, feasibility, and potential efficacy of cathodal tDCS to salvage the penumbra in acute large vessel occlusion (application before and after endovascular therapy) are expected in 2024 (TESSERACT-BA, ClinicalTrials.gov NCT04061577). However, since we and others have not found augmentation of neuroplastic processes both in human and rodent conditions by cathodal tDCS^18,23,24,51–54^, the current work focuses on safety of anodal tDCS which may be combined with interventions augmenting learning and re-learning after stroke in early neurorehabilitation.

### Implications for the application in the clinical setting

Direct translation of stimulation parameters derived from animal models to the human setting is certainly limited due to obvious reasons. Modelling data may give an idea of a scaling factor to compare current densities between species^35^. However, ipsilesional anodal tDCS in the stroke rat model appears to be safe as long as it is not applied during the hyperacute phase after stroke manifestation (within hours of stroke induction) and below a certain harmful threshold intensity. 24 h post-stroke this threshold intensity does not differ from that obtained in the healthy rat brain. Thus, we were able to exclude sensitization of the rat brain to increasing doses of tDCS by a recent stroke. Accordingly, the commonly used clinical tDCS stimulation parameters considered safe in healthy subjects are likely safe in the acute phase after stroke in the early neurorehabilitation setting.

### Supplementary Information

Supplementary Figure 1.

## Data Availability

The datasets generated and analyzed during the current study are available from the corresponding author on reasonable request.

## References

[1] Kwakkel G, Kollen B, Twisk J (2006). Impact of time on improvement of outcome after stroke. Stroke.

[2] Carmichael ST (2005). Rodent models of focal stroke: Size, mechanism, and purpose. NeuroRx.

[3] Krakauer JW (2006). Motor learning: its relevance to stroke recovery and neurorehabilitation. Curr. Opin. Neurol..

[4] Verheyden, G., Nieuwboer, A., De Wit, L., Thijs, V., Dobbelaere, J., Devos, H., *et al.* Time course of trunk, arm, leg, and functional recovery after ischemic stroke (2008). 10.1177/1545968307305456 17876069

[5] Hosp JA, Luft AR (2011). Cortical plasticity during motor learning and recovery after ischemic stroke.

[6] Murphy TH, Corbett D (2009). Plasticity during stroke recovery: From synapse to behaviour. Nat. Rev. Neurosci..

[7] Nudo RJ (2006). Mechanisms for recovery of motor function following cortical damage. Curr. Opin. Neurobiol..

[8] Diserens K, Rothacher G, Barnes M, Dobkin B, Bogousslavsky J (2005). Is early neurorehabilitation useful?. Recovery after Stroke.

[9] Bernhardt J, Godecke E, Johnson L, Langhorne P (2017). Early rehabilitation after stroke. Curr. Opin. Neurol..

[10] Coleman ER, Moudgal R, Lang K, Hyacinth HI, Awosika OO, Kissela BM (2017). Early rehabilitation after stroke: A narrative review. Curr. Atheroscler. Rep..

[11] Grefkes C, Grefkes C, Fink GR, Fink GR (2020). Recovery from stroke: Current concepts and future perspectives. Neurol. Res. Pract..

[12] Risedal A, Zeng J, Johansson BB (1999). Early training may exacerbate brain damage after focal brain ischemia in the rat. J. Cereb. Blood Flow Metab..

[13] Farrell R, Evans S, Corbett D (1979). Environmental enrichment enhances recovery of. Science.

[14] Kozlowski DA, James DC, Schallert T, Lawrence L, We F, Cody R (1996). Use-dependent exaggeration of neuronal injury after unilateral sensorimotor cortex lesions. J. Neurosci..

[15] Bindman LJ, Lippold OCJ, Redfearn JWT (1964). The action of brief polarizing currents on the cerebral cortex of the rat (1) during current flow and (2) in the production of long-lasting after-effects. J. Physiol..

[16] Nitsche MA, Paulus W (2001). Sustained excitability elevations induced by transcranial DC motor cortex stimulation in humans. Neurology..

[17] Nitsche MA, Paulus W (2000). Excitability changes induced in the human motor cortex by weak transcranial direct current stimulation. J. Physiol..

[18] Reis J, Schambra HM, Cohen LG, Buch ER, Fritsch B, Zarahn E (2009). Noninvasive cortical stimulation enhances motor skill acquisition over multiple days through an effect on consolidation. Proc. Natl. Acad. Sci. USA..

[19] Hamoudi M, Schambra HM, Fritsch B, Schoechlin-Marx A, Weiller C, Cohen LG (2018). Transcranial direct current stimulation enhances motor skill learning but not generalization in chronic stroke. Neurorehabil. Neural Repair..

[20] Allman C, Amadi U, Winkler AM, Wilkins L, Filippini N, Kischka U (2016). Ipsilesional anodal tDCS enhances the functional benefits of rehabilitation in patients after stroke. Sci. Transl. Med..

[21] Stagg CJ, Bachtiar V, O’Shea J, Allman C, Bosnell RA, Kischka U (2012). Cortical activation changes underlying stimulation-induced behavioural gains in chronic stroke. Brain..

[22] Elsner B, Kwakkel G, Kugler J, Mehrholz J (2017). Transcranial direct current stimulation (tDCS) for improving capacity in activities and arm function after stroke: A network meta-analysis of randomised controlled trials. J. Neuroeng. Rehabil..

[23] Fritsch B, Reis J, Martinowich K, Schambra HM, Ji Y, Cohen LG (2010). Direct current stimulation promotes BDNF-dependent synaptic plasticity: Potential implications for motor learning. Neuron..

[24] Podda MV, Cocco S, Mastrodonato A, Fusco S, Leone L, Barbati SA (2016). Anodal transcranial direct current stimulation boosts synaptic plasticity and memory in mice via epigenetic regulation of Bdnf expression. Sci. Rep..

[25] Jackson MP, Rahman A, Lafon B, Kronberg G, Ling D, Parra LC (2016). Animal models of transcranial direct current stimulation: Methods and mechanisms. Clin. Neurophysiol..

[26] Gellner AK, Reis J, Holtick C, Schubert C, Fritsch B (2020). Direct current stimulation-induced synaptic plasticity in the sensorimotor cortex: Structure follows function. Brain Stimul..

[27] Kim SJ, Kim BK, Ko YJ, Bang MS, Kim MH, Han TR (2010). Functional and histologic changes after repeated transcranial direct current stimulation in rat stroke model. J. Korean Med. Sci..

[28] Yoon KJ, Oh BM, Kim DY (2012). Functional improvement and neuroplastic effects of anodal transcranial direct current stimulation (tDCS) delivered 1 day vs. 1 week after cerebral ischemia in rats. Brain Res..

[29] Peruzzotti-Jametti L, Cambiaghi M, Bacigaluppi M, Gallizioli M, Gaude E, Mari S (2013). Safety and efficacy of transcranial direct current stimulation in acute experimental ischemic stroke. Stroke..

[30] Braun R, Klein R, Walter HL, Ohren M, Freudenmacher L, Getachew K (2016). Transcranial direct current stimulation accelerates recovery of function, induces neurogenesis and recruits oligodendrocyte precursors in a rat model of stroke. Exp. Neurol..

[31] Patel PM, Drummond JC, Cole DJ, Goskowicz RL (1995). Isoflurane reduces ischemia-induced glutamate release in rats subjected to forebrain ischemia. Anesthesiology..

[32] Razoux F, Garcia R, Léna I (2007). Ketamine, at a dose that disrupts motor behavior and latent inhibition, enhances prefrontal cortex synaptic efficacy and glutamate release in the nucleus accumbens. Neuropsychopharmacology..

[33] Mishima T, Nagai T, Yahagi K, Akther S, Oe Y, Monai H (2019). Transcranial direct current stimulation (tDCS) induces adrenergic receptor-dependent microglial morphological changes in mice. eNeuro..

[34] Gellner A-K, Reis J, Fritsch B (2016). Glia: A neglected player in non-invasive direct current brain stimulation. Front. Cell Neurosci..

[35] Bikson M, Grossman P, Thomas C, Zannou AL, Jiang J, Adnan T (2016). Safety of transcranial direct current stimulation: Evidence based update 2016. Brain Stimul..

[36] Watson BD, Dietrich WD, Busto R, Wachtel MS, Ginsberg MD (1985). Induction of reproducible brain infarction by photochemically initiated thrombosis. Ann. Neurol..

[37] Chen J, Sanberg PR, Li Y, Wang L, Lu M, Willing AE (2001). Intravenous administration of human umbilical cord blood reduces behavioral deficits after stroke in rats. Stroke..

[38] Whishaw IQ, Coles BLK (1996). Varieties of paw and digit movement during spontaneous food handling in rats: Postures, bimanual coordination, preferences, and the effect of forelimb cortex lesions. Behav. Brain Res..

[39] Allred RP, Adkins DAL, Woodlee MT, Husbands LC, Maldonado MA, Kane JR (2008). The Vermicelli Handling Test: A simple quantitative measure of dexterous forepaw function in rats. J. Neurosci. Methods..

[40] Schmued LC, Stowers CC, Scallet AC, Xu L (2005). Fluoro-Jade C results in ultra high resolution and contrast labeling of degenerating neurons. Brain Res..

[41] Schindelin J, Arganda-Carreras I, Frise E, Kaynig V, Longair M, Pietzsch T (2012). Fiji: An open-source platform for biological-image analysis. Nat. Methods..

[42] Winkler C, Reis J, Hoffmann N, Gellner A-K, Münkel C, Curado MR (2017). Anodal transcranial direct current stimulation enhances survival and integration of dopaminergic cell transplants in a rat Parkinson model. eNeuro..

[43] Rossi C, Sallustio F, Di Legge S, Stanzione P, Koch G (2013). Transcranial direct current stimulation of the affected hemisphere does not accelerate recovery of acute stroke patients. Eur. J. Neurol..

[44] Sattler V, Acket B, Raposo N, Albucher JF, Thalamas C, Loubinoux I (2015). Anodal tDCS combined with radial nerve stimulation promotes hand motor recovery in the acute phase after ischemic stroke. Neurorehabil. Neural Repair..

[45] Rueger MA, Keuters MH, Walberer M, Braun R, Klein R, Sparing R (2012). Multi-session transcranial direct current stimulation (tDCS) elicits inflammatory and regenerative processes in the rat brain. PLoS One..

[46] Jackson MP, Truong D, Brownlow ML, Wagner JA, McKinley RA, Bikson M (2017). Safety parameter considerations of anodal transcranial Direct Current Stimulation in rats. Brain Behav. Immun..

[47] Notturno F, Pace M, Zappasodi F, Cam E, Bassetti CL, Uncini A (2014). Neuroprotective effect of cathodal transcranial direct current stimulation in a rat stroke model. J. Neurol. Sci..

[48] Liu Y-H, Chan SJ, Pan H-C, Bandla A, King NKK, Wong PTH (2017). Integrated treatment modality of cathodal-transcranial direct current stimulation with peripheral sensory stimulation affords neuroprotection in a rat stroke model. Neurophotonics..

[49] Zhang K, Guo L, Zhang J, Rui G, An G, Zhou Y (2020). tDCS accelerates the rehabilitation of MCAO-induced motor function deficits via neurogenesis modulated by the Notch1 signaling pathway. Neurorehabil. Neural Repair..

[50] Pruvost-Robieux E, Benzakoun J, Turc G, Marchi A, Mancusi RL, Lamy C (2021). Cathodal transcranial direct current stimulation in acute ischemic stroke: Pilot randomized controlled trial. Stroke..

[51] Nitsche MA, Schauenburg A, Lang N, Liebetanz D, Exner C, Paulus W (2003). Facilitation of implicit motor learning by weak transcranial direct current stimulation of the primary motor cortex in the human. J. Cogn. Neurosci..

[52] Galea JM, Celnik P (2009). Brain polarization enhances the formation and retention of motor memories. J. Neurophysiol..

[53] Sun Y, Lipton JO, Boyle LM, Madsen JR, Goldenberg MC, Pascual-Leone A (2016). Direct current stimulation induces mGluR5-dependent neocortical plasticity. Ann. Neurol..

[54] Buch ER, Santarnecchi E, Antal A, Born J, Celnik PA, Classen J (2017). Effects of tDCS on motor learning and memory formation: A consensus and critical position paper. Clin. Neurophysiol..

