# Supplementary material for: Safety of ipsilesional anodal transcranial direct current stimulation in acute photothrombotic stroke: implications for early neurorehabilitation
Source: Sci Rep. 2024 Jan 30;14:2501. doi: 10.1038/s41598-024-51839-5 (PMC10827716; doi:10.1038/s41598-024-51839-5)
Supplement: Supplementary file 1 — Supplementary Figure 1. [file 41598_2024_51839_MOESM1_ESM.docx]

**Supplemental Information**

**
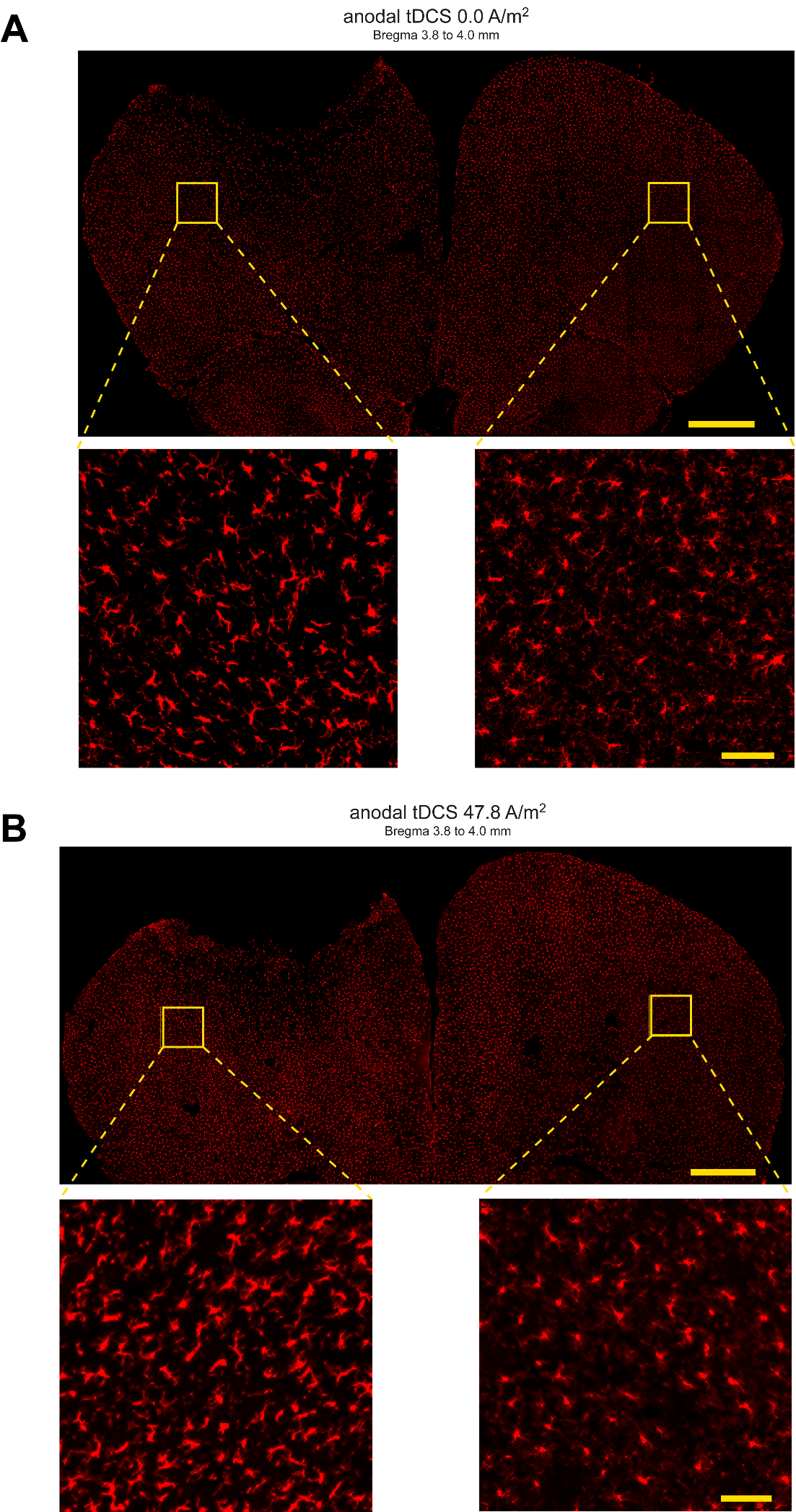
**

**Supplementary Figure 1:** Representative images of brain sections within the stroke region from animals treated with either (A) sham stimulation (0.0 A/m^2^) or (B) anodal tDCS of the highest intensity (47.8 A/m^2^). For each condition, overview pictures of the entire brain section and zoomed in selections from representative corresponding regions from the ipsi- and contralesional hemisphere are shown. Scale bar overview 1000 µm, scale bar zoomed in images 100 µm.
